# Comparative Analysis of Human Protein-Coding and Noncoding RNAs between Brain and 10 Mixed Cell Lines by RNA-Seq

**DOI:** 10.1371/journal.pone.0028318

**Published:** 2011-11-30

**Authors:** Geng Chen, Kangping Yin, Leming Shi, Yuanzhang Fang, Ya Qi, Peng Li, Jian Luo, Bing He, Mingyao Liu, Tieliu Shi

**Affiliations:** 1 Center for Bioinformatics and Computational Biology, and the Institute of Biomedical Sciences, College of Life Science, East China Normal University, Shanghai, China; 2 National Center for Toxicological Research, United States of America Food and Drug Administration, Jefferson, Arkansas, United States of America; 3 Shanghai Information Center for Life Sciences, Shanghai Institutes for Biological Sciences, Chinese Academy of Science, Shanghai, China; University of Münster, Germany

## Abstract

In their expression process, different genes can generate diverse functional products, including various protein-coding or noncoding RNAs. Here, we investigated the protein-coding capacities and the expression levels of their isoforms for human known genes, the conservation and disease association of long noncoding RNAs (ncRNAs) with two transcriptome sequencing datasets from human brain tissues and 10 mixed cell lines. Comparative analysis revealed that about two-thirds of the genes expressed between brain and cell lines are the same, but less than one-third of their isoforms are identical. Besides those genes specially expressed in brain and cell lines, about 66% of genes expressed in common encoded different isoforms. Moreover, most genes dominantly expressed one isoform and some genes only generated protein-coding (or noncoding) RNAs in one sample but not in another. We found 282 human genes could encode both protein-coding and noncoding RNAs through alternative splicing in the two samples. We also identified more than 1,000 long ncRNAs, and most of those long ncRNAs contain conserved elements across either 46 vertebrates or 33 placental mammals or 10 primates. Further analysis showed that some long ncRNAs differentially expressed in human breast cancer or lung cancer, several of those differentially expressed long ncRNAs were validated by RT-PCR. In addition, those validated differentially expressed long ncRNAs were found significantly correlated with certain breast cancer or lung cancer related genes, indicating the important biological relevance between long ncRNAs and human cancers. Our findings reveal that the differences of gene expression profile between samples mainly result from the expressed gene isoforms, and highlight the importance of studying genes at the isoform level for completely illustrating the intricate transcriptome.

## Introduction

Alternative splicing is a fundamental molecular process in eukaryotes, where it not only greatly increases the diversity of proteins that the genome can encode [1], but also contributes to the generating of long ncRNAs [2]. Exon skipping, mutually exclusive exons, intron retention, alternative donor and acceptor sites are five basic models of alternative splicing, beyond that, there are also other identified variations in splicing patterns [3], [4]. Individual mammalian genes often encode multiple different functional isoforms that may have related, distinct or even opposing functions through alternative splicing [5], [6]. A vast variety of gene isoforms generated by alternative splicing have specific roles in tissues or stages of development, and alterations in the RNA processing machinery may lead to mis-splicing of multiple transcripts and cause many diseases [7]–[9].

According to their protein-coding capacities, the gene isoforms could be divided into two distinct classes: messenger RNAs (mRNAs) which are translated into proteins, and the noncoding RNAs (ncRNAs), which function at the RNA level. Previous studies have indicated that most of the human genome is likely to be transcribed, generating a complex network of diverse transcripts that includes a large amount of ncRNAs [3], [10]. Those ncRNAs could be generated from diverse regions including intergenic, intronic areas, some of them have been proposed overlapping with protein-coding genes [11], [12]. Due to the majority of ncRNAs still have no clear significance in structure and lack strong sequence conservation, it has been suggested that they might be non-functional. In fact, a significant number of them have been shown to have important functions [13]–[18]. Those ncRNAs, which are recently identified and longer than 200 nt in length, are arbitrarily defined as the long ncRNAs [13], [14]. Previous researches have demonstrated that long ncRNAs can negatively [19], [20] or positively [21] affect the expression of neighboring protein-coding genes. It also has been proposed that a portion of long noncoding transcripts may be post-transcriptionally processed to generate small RNAs [22], such as microRNAs [23], [24], Piwi-interacting RNAs [25] and etc. Evidences have suggested that long ncRNAs are also associated with human diseases and could be used as cancer biomarkers, as well as therapy targets [26]–[29].

It is worth noticing that multi-exon genes could encode different isoforms through alternative splicing and different isoforms have different protein-coding potential. In addition, gene expression usually exhibit temporal and spatial specificity. Therefore, the same gene could generate different isoforms, even encoding both protein-coding and noncoding isoforms in different conditions or stages. RNA-Seq provides tremendous opportunities to reveal these diversities and the peculiar specificities of the human transcriptome [30]–[32]. Compared with microarrays, RNA-Seq can theoretically capture all the transcripts in the samples. Furthermore, RNA-Seq has low background noise, high sensitivity and requires less sample RNA [30], [31]. In principle, RNA-Seq can achieve single-base resolution, where microarrays rely on the density of probes. Besides, RNA-Seq can study gene expression at the isoform level but microarrays are unable to distinguish the isoforms and lost much valuable information related to the characteristics of the gene isoforms, such as the protein-coding capacity of each isoform, the number of expressed distinct isoforms, the composition and the expression level of each isoform.

To more comprehensively illustrate the complexity of human transcriptome, we investigated the diverse expression features of human known genes at both gene level and isoform level based on two RNA-Seq datasets generated from human brain tissues and 10 mixed cell lines. We first inferred the isoforms encoded in brain and cell lines, and then assessed the protein-coding capacities of all those expressed isoforms. Next, we compared the gene expression profile between brain and cell lines, and revealed that there are great differences in expression for those expressed genes between gene level and isoform level, implying that only the isoform level rather than gene level is able to accurately reflect the expression profiles of human genes. We also identified hundreds of long ncRNAs in brain or cell lines (total more than 1,000 long ncRNAs), and found a significant number of human genes in brain and cell lines could encode both protein-coding and noncoding RNAs through alternative splicing. Our results show that most long ncRNAs from brain and cell lines contain conserved elements across 46 vertebrates, or 33 placental mammalians, or 10 primates. Through RT-PCR we further validated that several long ncRNAs were differentially expressed in breast cancer cells versus normal breast cells, or between lung cancer cells and normal lung cells. In addition, those differentially expressed long ncRNAs are significantly correlated with a number of breast cancer or lung cancer related genes.

## Results

### Identifying the expressed isoforms in brain and cell lines

Our study used two transcriptome sequencing datasets from two reference RNA samples established by the MicroArray Quality Control (MAQC) project [33] with standard Illumina next-generation sequencing technology: the Universal Human Reference RNA (UHRR or UHR) from 10 human cell lines of various origins [34] and the Human Brain Reference RNA (HBRR or HBR) from several regions of the brain of 23 adult donors (see Materials and Methods). These two datasets consisted of ∼59.46 million and ∼53.24 million of sequencing reads, respectively, with read-lengths of 35 bp.

**Figure 1:**
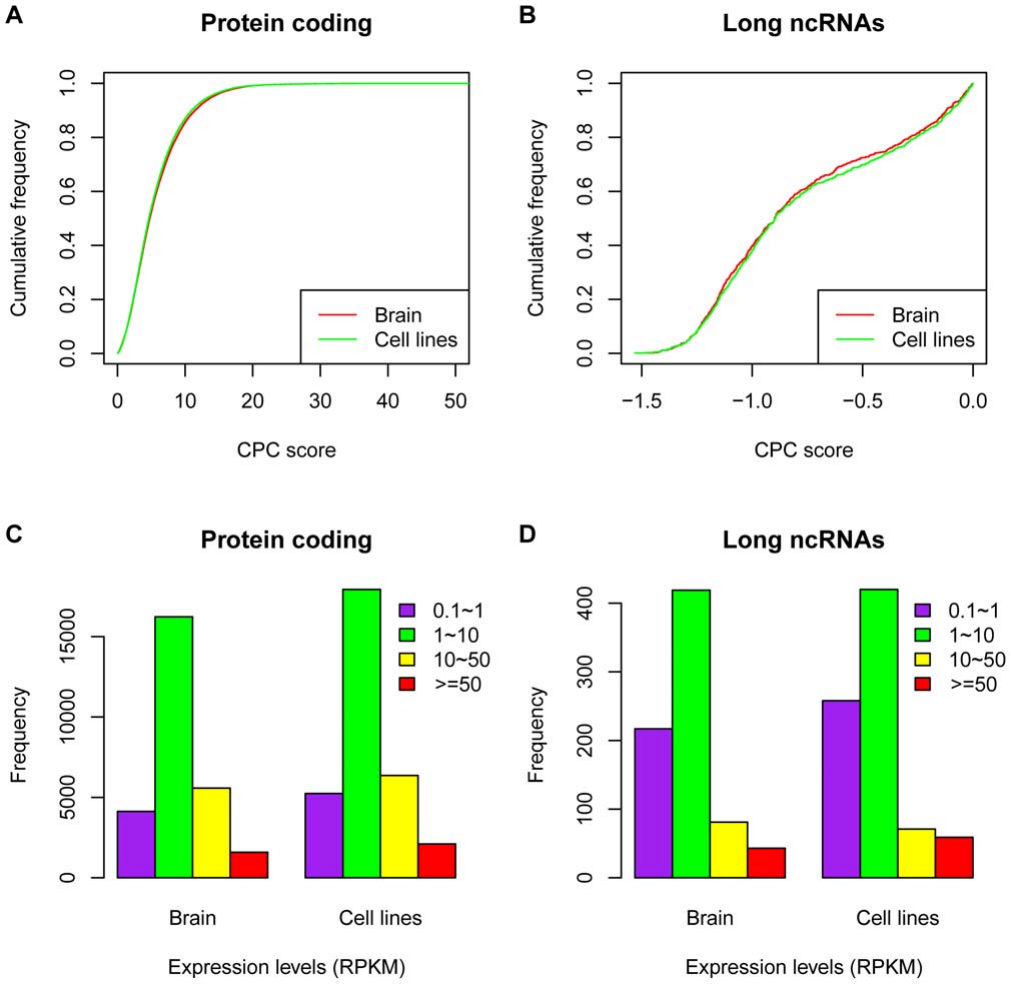
Protein coding capacity and expression of brain and cell line protein-coding transcripts and long ncRNAs. A, B are coding capacities of brain and cell line protein-coding transcripts and long ncRNAs, shown as the cumulative distribution of CPC scores. C, D are expression levels of brain and cell line protein-coding transcripts and long ncRNAs, shown as the bar plot distribution of expression levels, in reads per kilobase of exonic sequence per million aligned reads (RPKM).

We first mapped the brain and cell line reads onto the human reference genome (hg19) and junction sequences using bowtie [35] with two mismatches allowed (see Tables 1 and 2 in Document S1). Based on the short read mapping information and the human gene annotations of UCSC, we then inferred the expressed isoforms in brain and cell lines [36]. Because isoforms expressed at extremely low levels often cannot be reliably constructed, and might be artifacts, we used 0.1 RPKM (**R**ead **P**er **K**ilobase of transcript per **M**illion mapped reads) as threshold, and obtained a total of 28,571 transcripts in brain from 16,818 UCSC annotated genes and 32,714 transcripts in cell lines from 18,431 UCSC annotated genes (Files S1 and S2). Lower threshold could find more transcripts expressed, but it could also decrease the accuracy of identified expressed isoforms due to the read mapping uncertainty caused by the paralogous gene families, repeats and high sequence similarity between alternative spliced isoforms of the same gene [37]. Next, we assessed the protein-coding capacities of all brain and cell line transcripts using CPC (Coding Potential Calculator) [38]. In sum, 27,524 protein-coding and 1,047 noncoding RNAs were identified from the brain transcripts, while 31,641 protein-coding and 1,073 noncoding RNAs were found in cell lines (Figure 1). Among those noncoding RNAs, 760 (transcribed from 691 genes) in brain and 808 (transcribed from 735 genes) in cell lines are longer than 200 nt without protein-coding potentials, they were classified as the long ncRNAs, and 380 long ncRNAs are in common between brain and cell lines.

**Table 1:** Exon number of brain and cell line transcripts.

Items	Brain	Cell lines
Number of one exon transcripts	1,748	1,723
Number of two exon transcripts	2,281	2,429
Number of three exon transcripts	2,888	3,294
Number of four exon transcripts	3,048	3,526
Number of five exon transcripts	2,943	3,410
Number of six or more exon transcripts	15,663	18,332

### Characteristics of brain and cell line transcripts

We then investigated the number of exons contained in each transcripts and the number of isoforms encoded by each gene in brain and cell lines. About 93.88% transcripts in brain and 94.73% transcripts in cell lines contained two or more exons (Table 1). According to our selection criteria (isoform expression level ≥ 0.1 RPKM as expressed), we found 11,048 (65.69%) genes generated only one isoform in brain, and the rest expressed genes encoded two or more isoforms. Similarly, 11,697 (63.46%) genes in cell lines generated only one isoform, and the rest expressed genes encoded two or more isoforms (Table 2). The average number of isoforms each gene generated is 1.7 for brain and 1.77 for cell lines. The results suggest that the majority of human expressed genes encoded only one isoform with a relative higher level that can be detected, and most of those expressed transcripts comprise two or more exons.

**Table 2:** Isoforms of the expressed genes in brain and cell lines.

Items	Brain	Cell lines
Total number of expressed genes	16,818	18,431
Total number of isoforms	28,571	32,714
Number of protein-coding transcripts	27,524	31,641
Number of noncoding transcripts	1,047	1,073
Number of long ncRNAs	760	808
Number of genes generated one isoform	11,048	11,697
Number of genes generated two isoforms	2,933	3,334
Number of genes generated three isoforms	1,352	1,548
Number of genes generated four isoforms	699	839
Number of genes generated five isoforms	352	446
Number of genes generated six or more isoforms	434	567

In this table, only those isoforms with expression level equal or greater than 0.1 RPKM were took into account.

**Figure 2:**
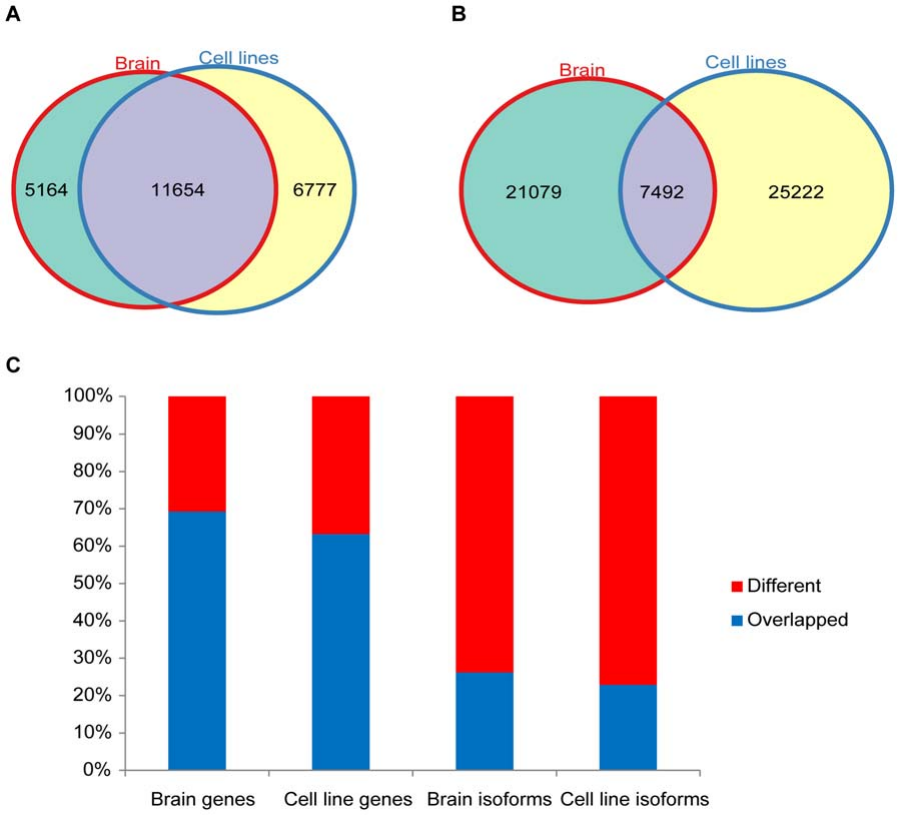
Comparison of the expression between brain and cell lines on the gene level and isoform level. A is the comparison in the number of expressed genes between brain and cell lines. B is the comparison in the number of expressed isoforms between brain and cell lines. C is the comparison in percentage between brain and cell line of expressed genes and isoforms.

In brain and cell lines, 11,654 expressed genes are in common, while 5,164 (30.71%) expressed genes only detected in brain and 6,777 (36.77%) expressed genes only detected in cell lines according to our threshold (Figure 2A). Interestingly, in those common expressed genes, only 3995 genes generated the equal number and identical isoforms in both brain and cell lines, the rest 7659 genes generated different isoforms. We also found that among those common expressed genes, 85 genes generated long ncRNAs only in brain but not in cell lines, whereas 117 genes generated long ncRNAs only in cell lines but not in brain; 33 genes encoded protein-coding RNAs only in brain, while 34 genes encoded protein-coding RNAs only in cell lines. Consequently, although most of the expressed genes in brain and cell lines are the same, in fact, only a minority of the expressed genes generated the same number and identical isoforms between brain and cell lines, leaving most of them different.

To further explore the different expression profiles between brain and cell lines, we compared the composition of all their expressed isoforms, and surprisingly found that only 7,492 isoforms (6,990 of them are protein-coding and 502 are noncoding) are the same between brain and cell lines, 21,079 (73.78%) isoforms (20,534 of them are protein-coding and 545 are noncoding) are expressed only in brain, 25,222 (77.1%) isoforms (24,651 of them are protein-coding and 571 are noncoding) are expressed only in cell lines (Figure 2B). About two-thirds of the expressed genes are in common between brain and cell lines, but at the isoform level, the result is reversed and only less than one-third of identical isoforms are expressed in both brain and cell lines (Figure 2C). Therefore, although the majority of expressed genes are the same between two samples, most of those common expressed genes encoded different isoforms. Moreover, the genes that only expressed in brain or cell lines directly contributed to the expression differences between them. These findings further uncover the complexity of transcriptional and post-transcriptional processing of the human transcriptome.

### Genes encoding both protein-coding and noncoding RNAs

Previous evidences have indicated that some genes can encode bifunctional RNAs, with their certain splicing isoforms acting as regulatory noncoding RNAs, and other specific splicing isoforms being translated into a protein, such as LXRB/LXRBSV isoform pair [39]. Our study also revealed that 146 and 185 genes (49 of them in common) encoded both protein-coding and noncoding RNAs in brain and cell lines, generated 176 long ncRNAs in brain and 217 long ncRNAs in cell lines (41 long ncRNAs are in common), respectively. For the rest of 16,672 expressed genes in brain (Table 2), 545 of them only produced long ncRNAs and 15,843 only produced protein-coding RNAs; for the rest of 18,246 expressed genes in cell lines, 550 of them only produced long ncRNAs and 17,438 only produced protein-coding RNAs. These results further demonstrate the dynamic expression patterns for transcripts in different samples, and suggest that isoform switching could grant the same gene with different roles according to the temporal and spatial specificity.

To better characterize the expression features of those long ncRNAs which transcribed from the 146 brain and 185 cell line genes that encode bifunctional RNAs, we further compared the exon structures of those long ncRNAs with their gene annotations on the reference genome. We found that 13 brain long ncRNAs contain introns, 79 brain long ncRNAs with new alternative exon boundaries or new exons and 84 brain long ncRNAs were produced by the exon skipping mechanism; 17 cell line long ncRNAs contain introns, 90 cell line long ncRNAs with new alternative exon boundaries or new exons and 110 cell lines long ncRNAs belong to the alternative splicing events of exon skipping. Those long ncRNAs which contain introns or with new alternative exon boundaries or new exons might result from the intron retention or alternative splice site usage or the incomplete annotation of their genes on the reference genome [2], [40]. Our findings confirm that a number of human known genes can encode both protein-coding and noncoding RNAs through alternative splicing, showing the importance of considering the protein-coding capacities of isoforms.

### Conservation and disease association of long ncRNAs

To gain insights into the evolutionary conservation of those long ncRNAs identified in brain and cell lines, we investigated the base-by-base phastCons scores (see Figure 1 in Document S1) and searched the phastCons-predicted conserved elements (see Figure 2 in Document S1) in each long ncRNA across 46 vertebrates, or their 33 placental mammal subset of species, or their 10 primate subset of species [41], respectively (see the ‘Conservation of long ncRNAs’ paragraph in Document S1). We found that a portion of long ncRNAs in human brain and cell lines are highly conserved among vertebrates or mammals or primates and most of those long ncRNAs contain conserved elements.

We further explored the expression profiles of long ncRNAs in human cancers. We first aligned the probes of Human Genome U133 Plus 2.0 Array from Affymetrix to the brain and cell line long ncRNAs to find whether any probe can represent the expression of our identified long ncRNAs. Using the exact and unique match criteria, 56 probes with no corresponding gene symbols could be aligned to 56 brain long ncRNAs and 59 probes for cell line long ncRNAs. Then we detected the differential expression of those probes in two human Gene Expression Omnibus (GEO) datasets, one is about breast cancer [42], another is about lung cancer [43]. We found that 10 brain and 9 cell line long ncRNAs differentially expressed in breast cancer, 17 brain and 11 cell line long ncRNAs differentially expressed in lung cancer, with 5 brain and 2 cell line long ncRNAs in common between breast and lung cancers.

**Figure 3:**
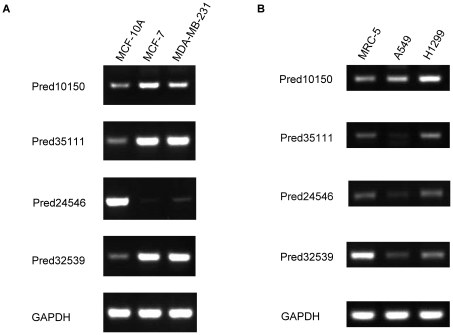
RT-PCR validation of the expression profiles of long ncRNAs in human diseases. A is the expression profiles of long ncRNAs in breast cancer cells (MCF-7, MDA-MB-231) and normal breast cells (MCF-10A). B is the expression profiles of long ncRNAs in lung cancer cells (A549, H1299: non-small cell lung carcinoma) and normal lung cells (lung fibroblast). GAPDH was used as an expression control. Four long ncRNAs are all differentially expressed in breast cancer versus normal breast cells, and two differentially expressed (“Pred10150” and “Pred32359”) between lung cancer and normal lung cells.

We then chose those five brain long ncRNAs (they are all from the genes that only generate long ncRNAs) which were differentially expressed in both breast cancer and lung cancer as the candidates to validate our results with RT-PCR. All but one long ncRNAs were obviously differentially expressed in breast cancer cells versus normal breast cells (three of them were up-regulation, one was down-regulation), two long ncRNAs (one was up-regulation and the other one was down-regulation) were apparently differentially expressed between lung cancer cells and normal lung cells (Figure 3). These results present the feasibility of our selected probes to represent our identified long ncRNAs to investigate their expression profiles in human diseases. We also found that those differentially expressed long ncRNAs have significant correlations (r≥0.4 and p<0.05) with some previously identified breast cancer or lung cancer related genes (Tables S1 and S2), such as long ncRNA “Pred35111” was significantly correlated with LEPR (r = 0.66, p =  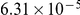), and LEPR has reported in several studies that it involves in the pathogenesis of breast cancer [44]–[47].

## Discussion

In this study, we investigated the diverse expression profiles of present human known genes based on two RNA-Seq datasets from human brain tissues and a variety of cell lines. We found that under our selection criteria, the majority of (more than 60%) the expressed genes in both brain and cell lines generated only one isoform with relative higher level that can be detected, whereas a portion of the expressed genes could encoded more than five isoforms. In those expressed genes of brain and cell lines, a number of them could encode both protein-coding and noncoding RNAs through alternative splicing, implicating the intricacy of gene transcription and post-transcriptional RNA processing. We also found that although most of the expressed genes in human brain and cell lines are the same, but their expressions are significantly different at the isoform level, and only less than 30% of the isoforms are identical between brain and cell lines. Besides that, some expressed genes only generated protein-coding or noncoding transcripts in one sample but not in another. Our results reveal that the significant difference for gene expression profiles between brain and cell lines is not only relevant to the sequence compositions of their isoforms, but also associated with the protein-coding capacities of their isoforms. Furthermore, when taking the expression level of each isoform into consideration, the variations for gene expression will inevitably increase more dramatically. Accordingly, only based on the isoform level, can we accurately recognize the gene expression pattern and estimate the gene expression profiles in various biological conditions and different tissues, such a fine scale will definitely improve our ability to gain new important insights into the functionalities for each gene on the human genome.

Therefore, to comprehensively explore the differences between different tissues, we need to consider isoforms the genes generated in addition to identify the expressed genes. Due to the complex alternative splicing in mammalians, the actual expression level of multi-exon genes is not only determined by the number of distinct isoforms they encoded, but also related to expression abundance of each isoform. Thus, the expression level of a gene might not be present differential expression in two samples, but it does not mean that this gene encodes the same isofoms in both samples. Because multi-exon genes could encode multiple different isoforms through alternative splicing and different isoforms would result in the same expression level of the gene between the two samples. Furthermore, the multi-exon genes would even encode both protein-coding and noncoding isoforms. Different isofroms of the same gene could play different roles with the temporal and spatial specificity [5], [6]. Consequently, the isoform level provides us more abilities to precisely determine the real gene expression patterns, and would help us to refine the gene regulation network and interaction models. However, the microarrays rely on prior information and cannot reflect the gene expression at the isoform level but rather at the gene level. Our study highlights the advantages of RNA-Seq studies over microarrays for the analysis of a comprehensive transcriptome.

We also found that the majority of brain and cell line long ncRNAs contain the regions similar to the phastCons-predicted conserved elements across 46 vertebrates, or their 33 placental mammal subset, or their 10 primate subset. Consequently, although the entire transcripts of most long ncRNAs lack strong conservation, yet highly conserved elements are preserved in them. In addition, some conserved element fragments of vertebrates, placental mammals and primates in the long ncRNAs are not similar to each other, suggesting that those conserved element fragments provide the structural base for the corresponding long ncRNAs to play special roles in vertebrates, placental mammals or primates. We also observed that some long ncRNAs are differentially expressed between breast cancer cells and normal breast cells, or between lung cancer cells and normal lung cells, and those differentially expressed long ncRNAs are significantly correlated with several identified breast cancer or lung cancer related genes. The results indicate that those differentially expressed long ncRNAs may directly or indirectly influence the expression of the cancer related genes. Our findings further imply that long ncRNAs could be involved in human cancers and play important roles in the pathogenesis of human diseases. In conclusion, these results indicate that long ncRNAs are not transcriptional “noise”, but might be functional macromolecules and they might have special relationship with cancer related genes.

Our study suggests that to comprehensively investigate the overall expression profiles of human genes, it is important to consider the following aspects: the number of distinct isoforms encoded by each gene; the protein-coding capability of each isoform; the composition of the isoforms generated by alternative splicing and the expression levels of those isoforms and etc. Although our comparison is based on human brain and 10 mixed cell lines, it is anticipated that the results could also be applied to the comparison among different tissues or cell lines. Current annotated genes on the human reference genome are mainly about protein-coding genes and known stable noncoding RNAs, such as tRNAs, snRNAs and snoRNAs, only a few of those annotated genes could encode long noncoding RNAs (some genes could encode both protein-coding and non-coding RNAs, but these genes could be annotated only as protein-coding genes). However, previous transcriptomics studies have indicated that a huge number of transcripts in mammals do not seem to encode proteins but function as noncoding RNAs instead [2], [48]. Hence a large amount of long ncRNAs transcribed from the intergenic regions still need to be identified. *Ab initio* reconstruction of the human transcriptome is crucial for the identification of those long ncRNAs which transcribed from the intergenic regions, With the improvements of sequencing technologies and the algorithms for *ab initio* reconstruction of the transcriptome, it will enable us more comprehensively and more thoroughly to comprehend the human transcriptome.

## Materials and Methods

### Data production

Two transcriptome sequencing datasets were generated from two reference RNA samples established by the US FDA-led MicroArray Quality Control (MAQC) project [33] with Illumina next-generation sequencing technology. The reference RNA sample A (UHRR, Catalog #740000) consists of total RNA extracted from 10 human cell lines of various origins: Blymphocyte, brain, breast, cervix, liposarcoma, liver, macrophage, skin, testis and Tlymphocyte [34]. Equal quantities of DNAase-treated total RNA from each cell line were pooled to generate the UHRR. The reference RNA sample B (HBRR, Catalog #6050) consists of total RNA extracted from several regions of the brains from 23 adult donors.

These two samples were prepared using the standard Illumina mRNA-Seq protocol and reagents. The sequencing data were single end reads, with ∼59.46 million and ∼53.24 million of sequencing reads in 35 bp length for cell line and brain samples, respectively. Those two datasets from this study have been submitted to the NCBI Gene Expression Omnibus (http://www.ncbi.nlm.nih.gov/geo) under accession number GSE30222.

### Identification of isoforms and assessing the protein-coding capacities of isoforms

The brain and cell line reads were first aligned onto the human reference genome hg19 (http://genome.ucsc.edu/) and junction sequences using bowtie [35] with default parameters (allowed two mismatches). Then we used those mapping information to infer the isoforms transcribed from brain and cell lines base on the human gene annotation information of UCSC by isoinfer [36].

Next, we calculated the protein-coding capacities of all brain and cell line transcripts using CPC [38] which incorporates the sequence features into a support vector machine to assess the protein-coding potential of each transcript. According to the CPC scores, we split the brain and cell line transcripts into protein-coding and noncoding two classes. Then those transcripts which are longer than 200nt and lack protein-coding potential were classified as the long ncRNAs, and were used for the further analysis.

### Calculation of the conservation of long ncRNAs

We separately downloaded the base-by-base phastCons [41] scores and the phastCons-predicted conserved elements across the 46 vertebrates, and the 33 placental mammal subset of species in these 46 vertebrates and the 10 primate subset of species in these 46 vertebrates from UCSC (http://genome.ucsc.edu/). The phastCons scores can be interpreted as probabilities that each base is in a conserved element, given the assumptions of the model and the maximum-likelihood parameter estimates [41]. We first calculated the base conservation in each long ncRNAs using the criteria that the phastCons score of base is not less than 0.9, and then we calculated the ratio of each brain or cell line long ncRNA transcript overlapping the phastCons-predicted conserved elements across 46 vertebrates or 33 placental mammals or 10 primates.

### Detecting differentially expressed long ncRNAs and RT-PCR validation

To investigate the disease association between long ncRNAs and human cancers, we first aligned the probes of Human Genome U133 Plus 2.0 Array onto the brain long ncRNAs to find the probes that can represent the expression of our identified long ncRNAs using the exact and unique match criteria. Then we detected the differential expression of those probes in two human Gene Expression Omnibus (GEO) datasets: GSE10810 which was about breast cancer [42] and GSE10799 which was about lung cancer [43]. We further checked the selected candidate differentially expressed long ncRNAs using RT-PCR to validate whether they are truly differentially expressed in breast cancer cells (MCF-7, MDA-MB-231) versus normal breast cells (MCF-10A), and between lung cancer cells (A549, H1299: non-small cell lung carcinoma) and normal lung cells (lung fibroblast). Primers were provided in Table 3 in Document S1).

## Supporting Information

Document S1 **This file records all the related supporting information mentioned in the main text.**
(DOC)

Table S1 **Significantly correlated genes of the long ncRNAs that differentially expressed in breast cancer.**
(XLS)

Table S2 **Significantly correlated genes of the long ncRNAs that differentially expressed in lung cancer.**
(XLS)

File S1 **Transcript expression values, coordinates and protein-coding capacities in brain.**
(TXT)

File S2 **Transcript expression values, coordinates and protein-coding capacities in cell lines.**
(TXT)
