# Supplementary material for: Comparative Analysis of Human Protein-Coding and Noncoding RNAs between Brain and 10 Mixed Cell Lines by RNA-Seq
Source: PLoS One. 2011 Nov 30;6(11):e28318. doi: 10.1371/journal.pone.0028318 (PMC3227660; doi:10.1371/journal.pone.0028318)
Supplement: Document S1 — This file records all the related supporting information mentioned in the main text. (DOC) [file pone.0028318.s001.doc]

**Supplemental information**

**Conservation of long ncRNAs**

We first calculated the base conservation in each long ncRNAs using the criteria that the phastCons score of a base is not less than 0.9, and found that most long ncRNAs in brain (83.16% in 46 vertebrates, 84.61% in placental mammalians, 89.47% in primates) and cell lines (82.43% in 46 vertebrates, 84.28% in placental mammalians, 89.73% in primates) are less than 0.4 (<40%) with this base ratio, whereas only a small portion of long ncRNAs are higher than 0.6 in brain (7.24% in 46 vertebrates, 6.45% in placental mammals, 3.95% in primates) and cell lines (7.92% in 46 vertebrates, 6.93%% in placental mammals, 4.33% in primates) (Supplementary Figure S1).

Compared with the phastCons-predicted conserved elements, we found that 656 (86.32%) of 760 long ncRNAs in brain contain conserved regions across 46 vertebrates (exon overlapping length: 2 bp ~ 1004 bp); 611 (80.39%) have the regions conserved across the 33 placental mammals (exon overlapping length: 2 bp ~ 828 bp) while 455 (59.87%) possess conserved elements across the 10 primates (exon overlapping length: 3 bp ~ 1631 bp). In cell lines, among 808 long ncRNAs, 684 (84.65%) hold conserved regions across 46 vertebrates (exon overlapping length: 2 bp ~ 866 bp); 652 (80.69%) retain elements conserved across the 33 placental mammals (exon overlapping length: 2 bp ~ 801 bp) and 493 (61.01%) maintain conserved regions across the 10 primates (exon overlapping length: 4 bp ~ 1794 bp). Interestingly, among those conserved element fragments in brain and cell line long ncRNAs, some only appear in placental mammals or primates. Those diverse conserved element fragments might reflect their evolutionary history and grant the specific long ncRNAs special functions in placental mammals and primates.

We also calculated the lengths of the conserved regions to the fully length of each brain or cell line long ncRNA transcript according to the phastCons-predicted conserved elements (Supplementary Figure S2). Our results show that although the ratio is generally low for the majority of brain and cell line long ncRNAs, some brain and cell line long ncRNAs contain the high ratio of the conserved regions. This ratio of 86 (11.32%) brain and 101 (12.5%) cell line long ncRNAs are greater than 0.6 for the 46 vertebrate phastCons-predicted conserved elements; 78 (10.26%) brain and 85 (10.52%) cell line long ncRNAs are greater than 0.6 for the 33 placental mammal phastCons-predicted conserved elements; 78 (10.26%) brain and 79 (9.78%) cell line long ncRNAs are greater than 0.6 for 10 primate phastCons-predicted conserved elements.

**Supplemental Figures**


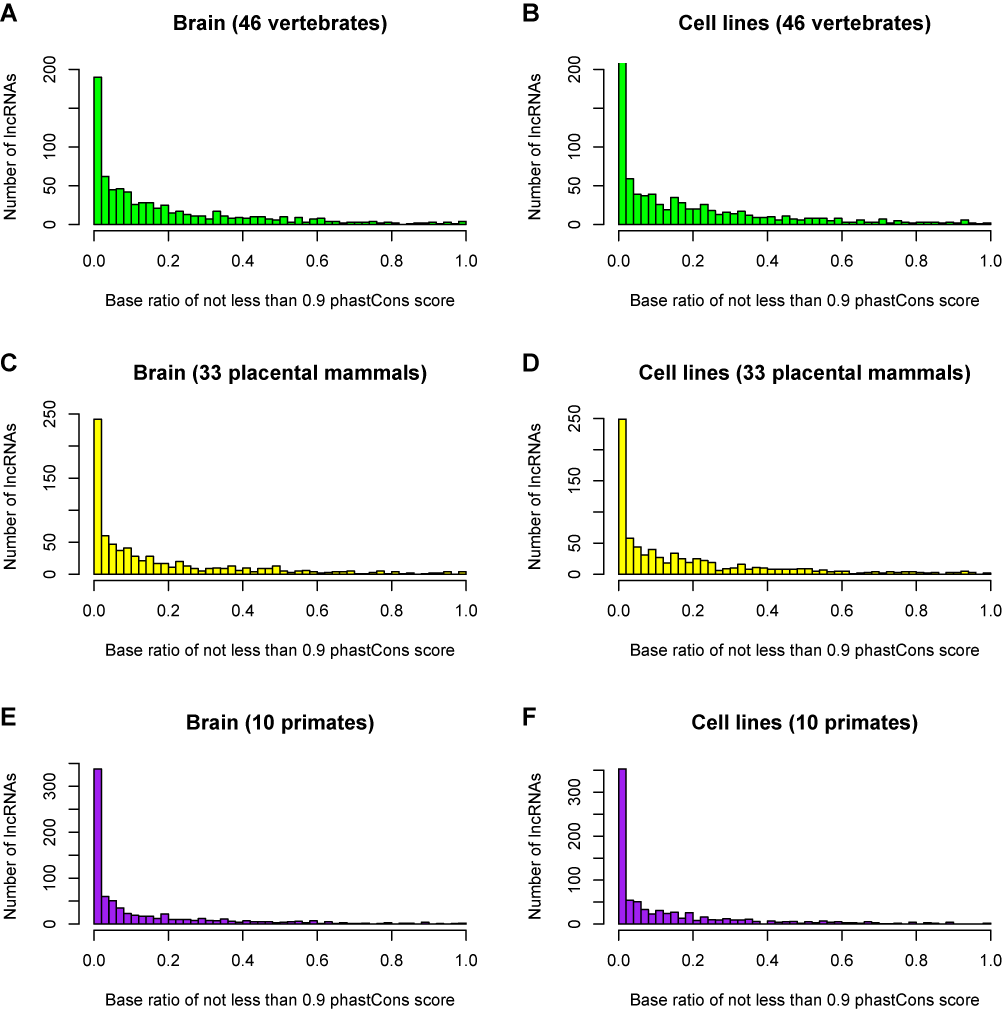


**Figure 1.** Base-by-base phastCons score of brain and cell line long ncRNAs. A, B, C, D, E, F are the base ratio of base phastCons score not less than 0.9 of brain and cell line long ncRNAs. Shown is the histogram distribution of base ratio of each long ncRNA transcript in which base phastCons score is not less than 0.9 across the 46 vertebrates, or the 33 placental mammals in those 46 vertebrates, or the 10 primates in those 46 vertebrates.

**
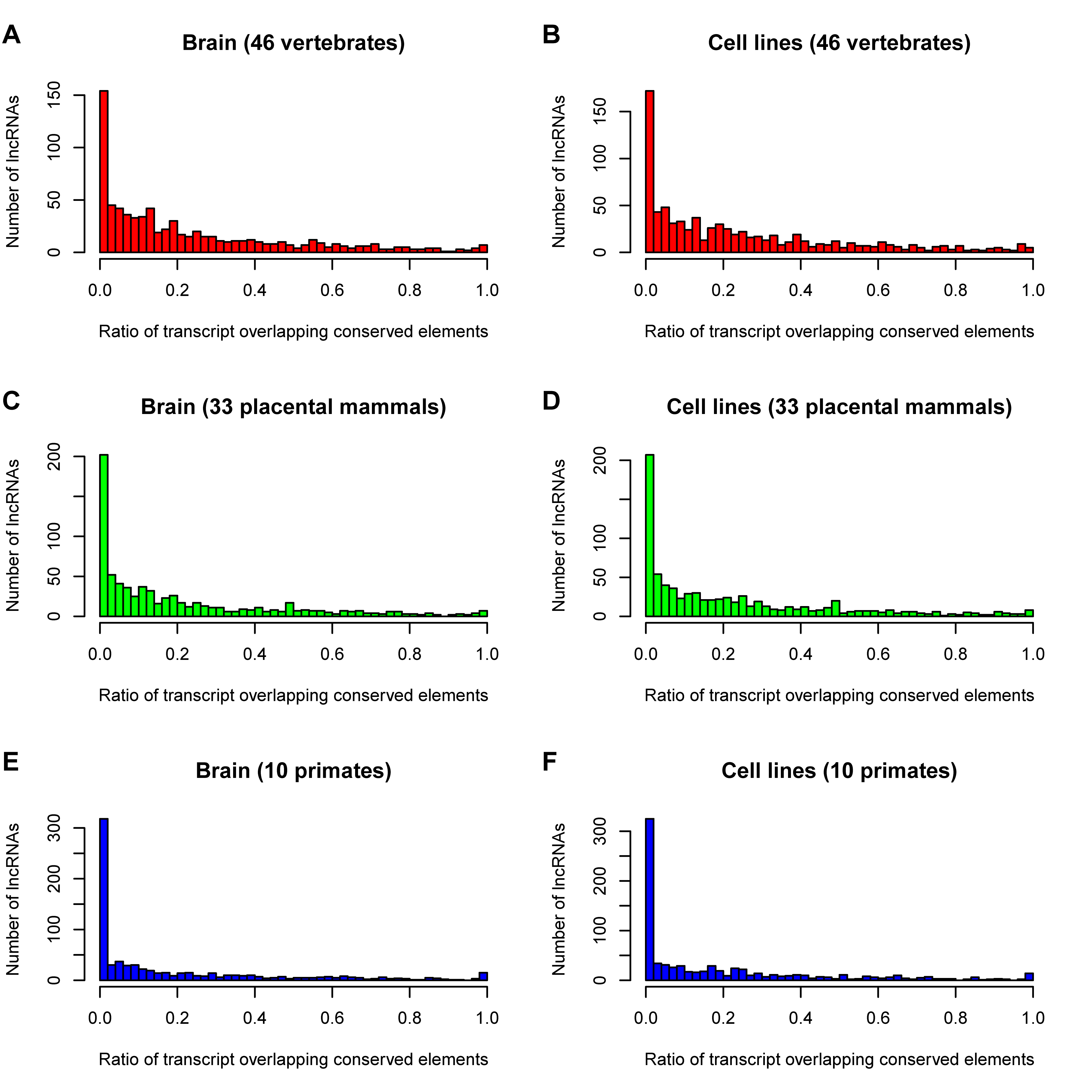
**

**Figure 2.** Sequence conservation of brain and cell line long ncRNAs. A, B, C, D, E, F are overlaps between phastCons-predicted conserved elements and brain or cell line long ncRNAs exons. Shown is the histogram distribution of the ratio of each brain or cell line long ncRNA transcript that overlap the phastCons-predicted conserved elements across 46 vertebrates, or their 33 placental mammal subset of species,or their 10 primate subset of species.

**Supplemental Tables**

**Table 1. Statistic of brain and cell line RNA-Seq reads mapped to hg19.**

| **Samples** | **Number of total reads** | **Number of mapped reads** | **Number of exact match reads** | **Number of one mismatch reads** | **Number of two mismatch reads** |
| --- | --- | --- | --- | --- | --- |
| Brain | 53,238,798 | 47,338,425 | 37,707,383 | 7,014,214 | 2,616,828 |
| Cell lines | 59,461,348 | 52,437,724 | 41,339,575 | 7,835,065 | 3,263,084 |

**Table 2. Statistic of brain and cell line RNA-Seq reads mapped to junction sequences.**

| **Samples** | **Number of total reads** | **Number of mapped reads** | **Number of exact match reads** | **Number of one mismatch reads** | **Number of two mismatch reads** |
| --- | --- | --- | --- | --- | --- |
| Brain | 53,238,798 | 4,763,706 | 3,339,174 | 675,201 | 749,331 |
| Cell lines | 59,461,348 | 7,025,573 | 5,248,765 | 1,035,308 | 741,500 |

**Table 3.** Validated differentially expressed long ncRNAs and their corresponding probes and primers.

| **Long ncRNAs** | **Probes** | **Primers** |
| --- | --- | --- |
| Pred10150 | 227332_at | s: 5' GGCTCTTTGTCTTCATCGT 3'  a: 5' CACTTCGTCTGTGCCTTCT 3' |
| Pred35111 | 229319_at | s: 5' CAGGGATAATGATGAAAGG 3'  a: 5' GATGCAGTCACCTCCTACC 3' |
| Pred24546 | 1557617_at | s: 5' CAGTAAGCAGTAGAAGGGAT 3'  a: 5' GAAATGGGAGGAGTAAGAC 3' |
| Pred32539 | 1560359_at | s: 5' CCCACTACTGTTCTTATGC 3'  a: 5' AAATGCTAGGGAGTCTTGT 3' |
